# Ribosomal MLST nucleotide identity (rMLST-NI), a rapid bacterial species identification method: application to *Klebsiella* and *Raoultella* genomic species validation

**DOI:** 10.1099/mgen.0.000849

**Published:** 2022-09-13

**Authors:** James E. Bray, Annapaula Correia, Margaret Varga, Keith A. Jolley, Martin C. J. Maiden, Charlene M. C. Rodrigues

**Affiliations:** ^1^​ Department of Zoology, University of Oxford, Oxford, UK; ^2^​ Department of Veterinary Medicine, University of Cambridge, Cambridge, UK; ^3^​ Wellcome Sanger Institute, Wellcome Genome Campus, Hinxton, UK; ^4^​ Department of Infection Biology, London School of Hygiene and Tropical Medicine, London, UK; ^5^​ Department of Paediatrics, Imperial College Healthcare NHS Trust, London, UK

**Keywords:** *Klebsiella*, *Raoultella*, bacterial species identification, rMLST, nucleotide identity, bacterial genomes

## Abstract

Bacterial genomics is making an increasing contribution to the fields of medicine and public health microbiology. Consequently, accurate species identification of bacterial genomes is an important task, particularly as the number of genomes stored in online databases increases rapidly and new species are frequently discovered. Existing database entries require regular re-evaluation to ensure that species annotations are consistent with the latest species definitions. We have developed an automated method for bacterial species identification that is an extension of ribosomal multilocus sequence typing (rMLST). The method calculates an ‘rMLST nucleotide identity’ (rMLST-NI) based on the nucleotides present in the protein-encoding ribosomal genes derived from bacterial genomes. rMLST-NI was used to validate the species annotations of 11839 publicly available *Klebsiella* and *Raoultella* genomes based on a comparison with a library of type strain genomes. rMLST-NI was compared with two whole-genome average nucleotide identity methods (OrthoANIu and FastANI) and the *k*-mer based Kleborate software. The results of the four methods agreed across a dataset of 11839 bacterial genomes and identified a small number of entries (*n*=89) with species annotations that required updating. The rMLST-NI method was 3.5 times faster than Kleborate, 4.5 times faster than FastANI and 1600 times faster than OrthoANIu. rMLST-NI represents a fast and generic method for species identification using type strains as a reference.

## Data Summary

An interactive BIGSdb project database containing bacterial genomes and provenance and phenotype information (meta-data) used in this study is available online (https://pubmlst.org/projects/klebsiella). Accession numbers of all genomes extracted from the NCBI Assembly database are listed within the individual isolate records of the BIGSdb project database and in the Supplementary Data Excel file (available in the online version of this article). The rMLST nucleotide identity (rMLST-NI) method is available as a webserver for searching genome sequences against 17 *Klebsiella* and *Raoultella* type strain genomes (https://pubmlst.org/species-id/klebsiella). The Multilocus Sequence Search (MLSS) software for performing rMLST-NI analyses is available on GitHub under the GNU General Public License v3.0 (https://github.com/jamesbrayuk/mlss DOI: 10.5281/zenodo.6514907). MLSS software requires NCBI blast (https://blast.ncbi.nlm.nih.gov) (version 2.12.0+or higher) and includes installation instructions and a tutorial with an example genome.

The allelic profiles of 17 *Klebsiella/Raoultella* type strains, DNA sequences of associated rMLST alleles and the rMLST-NI thresholds from this study and those implemented on the webserver are available on the webserver homepage. The DNA sequences of all rMLST alleles are also available via the online rMLST sequence definition database (https://pubmlst.org/species-id). Access to the rMLST sequence definition database requires user registration and is free for academic use.

**Impact Statement**

Accurate bacterial species identification is important in medical microbiology to understand which pathogens are responsible for causing disease. Increasingly, diagnostic tests in hospitals are performed using DNA-based tools that work by identifying DNA that is unique to specific bacterial species. Computational scientists study the DNA found in known bacterial samples available in large online genome databases to identify which DNA sequences can be used for diagnostic tests and other applications. It is therefore important that the species labels of the known bacterial samples are regularly re-evaluated and updated, for these computational analyses to yield accurate results. We have developed a new method, rMLST nucleotide identity (rMLST-NI), to validate species annotations and demonstrated its use with *Klebsiella/Raoultella* genomes using type strain genomes as the reference point. We applied the method to a large dataset of *Klebsiella/Raoultella* genomes (>11000) and identified 89 incorrect annotations. The MLSS.pl software for calculating rMLST-NI is 3.5 times faster than Kleborate and 4.5 times faster than the alignment-free FastANI approach. The rMLST-NI method uses the DNA sequences of genes that encode ribosomal proteins. As these genes are present in all bacteria, this approach can be further developed to rapidly validate the species annotations of many other bacterial pathogens and therefore has wide application and interest.

## Introduction

In the first decades of the 21^st^ century, microbial genomics made increasingly important contributions to the fields of clinical microbiology and infectious disease. The identification of pathogens for diagnosis, surveillance, and outbreak investigation are all enhanced with the use of high-resolution data obtained from bacterial whole-genome sequencing (WGS). To facilitate these applications, computational approaches have been developed that exploit the large volume of WGS available in online databases. Such approaches can identify which DNA sequences can be used to develop methods for diagnostic testing and other patient-facing applications. It is therefore imperative that the species annotations in these databases are continually checked and updated, in light of changes in taxonomic nomenclature and an understanding of bacterial population structure.

Since novel bacterial species evolve through genetic diversification, the comparison of bacterial genomes can establish how closely two variants are related and/or their relationship to a species reference genome, derived from a previously defined type strain. Genomic techniques such as average nucleotide identity (ANI) are used extensively for identifying novel species in the field of taxonomy, by calculating the nucleotide identity across the whole genome with type strains in the same genus or family [1–3]. At the time of writing, alignment-based ANI algorithms were computationally intensive and therefore had not been widely adopted in research or clinical settings for performing high-throughput whole-genome DNA comparisons. Alignment-free algorithms have been shown to be a computationally more efficient method to perform these calculations and can be applied to large genomic datasets [3]. An alternative approach to ANI calculates the similarity between genomes based on a small subset of universal genes [4, 5]. Ribosomal MLST (rMLST), is a universal, bacterial domain-wide approach that indexes the protein-encoding genes of the ribosome and has been shown to reconstruct phylogenetic and taxonomic groups accurately [6]. These genes can be identified in a computationally efficient manner using the BIGSdb (Bacterial Isolate Genome Sequence Database) platform for bacterial genome analysis [7]. The rMLST profiles are defined based on the numeric allelic indices of the 53 rMLST loci and are annotated with taxonomic information that has been verified by phylogenetic analyses. Phylogenetic trees can be reconstructed based on concatenating the rMLST allelic sequence variants that have been extracted from each genome and are visually inspected to assess the clustering of a query genome relative to a set of type strains. This approach has been used alongside ANI to define species within the *Neisseria* genus [8] and to validate the species annotations of *Campylobacter jejuni/coli* isolates [9]. There are rMLST profiles for 14161 bacterial species available to date.

The genus *Klebsiella* consists of 13 species of Gram-negative, oxidase-negative bacteria (Table 1) that are found ubiquitously in the environment, colonizing plants, animals and humans [10, 11]. Exposure to *Klebsiella* species can result in infections in healthy individuals, generally with hypervirulent strains, or in immunocompromised hosts, usually with ‘opportunistically’ or ‘accidentally’ invasive variants. Several *Klebsiella* species have become increasingly antibiotic resistant and at the time of writing were one of the World Health Organization’s (WHO) critical priority pathogens [12] and responsible for multi-drug-resistant nosocomial infectious disease outbreaks [13–18] with substantial impact on morbidity and mortality. *Klebsiella* spp. demonstrate overlapping phenotypes and can therefore be difficult to distinguish by conventional microbiological and biochemical methods. Four species in the *Raoultella* genus are closely related to *Klebsiella* [19]. These overlapping phenotypes and closely related genotypes can contribute to the mis-annotation of species that are entered into genomic databases. Further, there were nine new species within the *Klebsiella/Raoultella* genera identified between 2013 and the time of writing (Table 1), demonstrating that the species annotations of existing records in online genome databases inevitably become outdated as new knowledge is acquired. With more than 11000 *Klebsiella/Raoultella* bacterial genomes currently available in the NCBI Assembly database (https://www.ncbi.nlm.nih.gov/assembly) at the time of writing, performing a large-scale retrospective species validation analysis accurately and efficiently represented a significant computational challenge. However, it is important that the species labels of these genomes are kept up to date so that computational analyses that use this genomic data can be performed accurately, e.g. to develop diagnostic testing methods, perform bacterial surveillance or outbreak analysis.

**Table 1.** Summary of the 17 species in the *Klebsiella* and *Raoultella* genera with whole-genome sequences available at the NCBI Assembly database on 5th March 2021

* Klebsiella pneumoniae* species complex	
* K. pneumoniae* (Kp1)	* K. pneumoniae* are present in the human microbiota of the gut, nose, mouth and skin, and are the most clinically important *Klebsiella* species [32]. These bacteria can cause invasive disease, particularly in immunocompromised hosts as an opportunistic pathogen causing pneumonia, urinary tract infections and soft tissue infections [33].
* K. quasipneumoniae* subsp. *quasipneumoniae* (Kp2), *K. quasipneumoniae s* ubsp. *similipneumoniae* (Kp4)	* K. quasipneumoniae* was defined as a novel species in 2014 from an analysis of human blood culture isolates from European countries. The type strain isolate 01A030^T^ was isolated in 1997 from Austria [34].
* K. variicola* subsp. *variicola* (Kp3), *K. variicola* subsp. *tropica* (Kp5)	* K. variicola* colonise plants (rice, sugar cane, banana), insects, animals and humans and were identified as a species separate from *K. pneumoniae* in 2004 [35]. *K. variicola* can cause disease in animals (e.g. bovine mastitis [36]) and humans [37].
*K. quasivariicola* (Kp6)	*K. quasivariicola* was identified from a collection of extended spectrum beta-lactamases (ESBL) *Klebsiella* spp. isolates from patient samples in the United States and defined as a novel species in 2017 [38].
* K. africana* (Kp7)	* K. africana* (originally named *K. africanensis *) was defined in 2019 and these bacteria have been identified in human faecal carriage samples from Senegal (sampled in 2016) and in human clinical isolates from Kenya [24].
**Other species** (listed alphabetically)	
* K. aerogenes *	* K. aerogenes* can cause opportunistic infections in hospital settings [39]. The species was added to the *Klebsiella* genus in 2017 [40] (originally known as *Aerobacter aerogenes*, and later as *Enterobacter aerogenes *).
* K. grimontii* (Ko6)	* K. grimontii* was defined in 2018 after analysing isolates from human infections (blood cultures, superficial infections, haemorrhagic colitis) and human faecal carriage [28].
* K. huaxiensis* (Ko8)	* K. huaxiensis* was defined in 2019 from bacteria isolated from a human urinary tract infection in a hospitalized patient in China [41].
* K. indica *	* K. indica* was defined in 2020 from the surface of a tomato collected in a vegetable market in India (sampled in 2014) [42].
* K. michiganensis* (Ko1)	* K. michiganensis* was defined in 2013 following the analysis of a sample collected from the surface of a toothbrush holder in 2010 during a microbial ecology study in a household in Michigan [27].
* K. oxytoca* (Ko2)	* K. oxytoca* was first described in 1886 from sour milk and named *Bacillus oxytocus perniciosus*. *K. oxytoca* infections often occur in hospital environments and are considered opportunistic, occurring in immunosuppressed hosts and causing a range of infections including pneumonia, urinary tract infections and soft tissue/wound infections [43].
* K. pasteurii* (Ko4)	* K. pasteurii* was identified as a novel species in 2019 from the faecal samples from humans, cows and turtles collected in Italy in 2017/2018 [23].
* K. spallanzanii* (Ko3)	* K. spallanzanii* was identified as a novel species in 2019 from samples of human urine and bovine faeces collected in Italy in 2017/2018 [23].
* Raoultella electrica *	* R. electrica* was defined as a novel species in 2014 from biofilms found on a glucose-fed microbial fuel cell in Japan (sampled in 2013) [44].
* Raoultella ornithinolytica *	* R. ornithinolytica* are found in aquatic environments and fish. They are increasingly recognised as a cause of human opportunistic infections with a broad spectrum including: bone, biliary tract, urinary tract, intracranial, pneumonia and bloodstream infections [45].
* Raoultella planticola *	* R. planticola* are naturally occurring in environmental niches including soil and water [46]. They are emerging as a recognized cause of invasive disease, largely in immunocompromised hosts.
* Raoultella terrigena *	* R. terrigena* are naturally occurring in environmental niches including soil and water [47]. They are rarely responsible for causing human disease.

This study extended the rMLST phylogenetic approach to calculate the similarity of a query genome automatically and rapidly to a library of type strain genomes using ribosomal allelic variants and determine the level of similarity required to reliably assign species annotations. We developed an efficient method to calculate the rMLST nucleotide identity (rMLST-NI) between a query genome and a library of type strain genomes described by a set of rMLST profiles and pre-defined rMLST allele sequences. Importantly, this method is generic to the bacterial domain and could be used for any species with a defined type strain genome and curated rMLST allele sequences. Here, the rMLST-NI method was directly applied to the large-scale retrospective validation of more than 11000 species annotations of *Klebsiella/Raoultella* genomes from the NCBI Assembly database (https://www.ncbi.nlm.nih.gov/assembly). The rMLST-NI results were compared against three automated species identification methods: (i) whole-genome ANI using OrthoANIu software [20, 21]; (ii) whole genome ANI using FastANI [3]; and (iii) Kleborate software species scan, a community-maintained resource (https://github.com/katholt/Kleborate) [22].

## Methods

### Genomic dataset creation

The dataset of *Klebsiella* and *Raoultella* genomes was created from information held in the NCBI Assembly database on 5 March 2021 (https://www.ncbi.nlm.nih.gov/assembly). Entries with a species annotation containing *Klebsiella* or *Raoultella* were identified and those entries with species-specific taxonomic annotations retained. The GenBank assembly accession, species annotation, BioSample and BioProject accessions, and type strain information were all extracted from the database. Entries labelled as ‘Excluded from RefSeq’ were removed from the dataset except those marked as ‘derived from environmental source’, to remove anomalous assemblies and avoid reporting issues that have already been identified by the NCBI annotation process (https://www.ncbi.nlm.nih.gov/assembly/help/anomnotrefseq). The dataset was not filtered to exclude any entries based on genome size, number of contigs or N50. A final list of 11839 GenBank assembly accessions was compiled, and genome sequences were downloaded from the NCBI ftp site (ftp://ftp.ncbi.nlm.nih.gov, links to individual genome sequences are included in the Supplementary Data Excel Spreadsheet).

### Genomic database creation and population

A Bacterial Isolate Genome Sequence Database (BIGSdb) project and website were created to manage the genomic data and associated provenance meta-data for this study (https://pubmlst.org/projects/klebsiella). The 11839 isolate records were created, and genomes uploaded to the appropriate BIGSdb isolate entry, based on the NCBI GenBank assembly accession. The BIGSdb project database website facilitates the exploration and export of genomic and isolate meta-data (e.g. genome size per species, Fig. S1). Species annotation changes were made to 18 database entries based on the species definitions in two publications from 2019 [23, 24] (Table S1). The BioSample records associated with these publications were updated at the European Nucleotide Archive (ENA, https://www.ebi.ac.uk/ena) before 5 March 2021, but the changes were not reflected at the NCBI Assembly database until after this date (Carla Rodrigues, Institut Pasteur, personal communication). WGS data from 17 type strain isolates were identified from the NCBI Assembly records and cross-referencing this with the defined species for *Klebsiella/Raoultella* in the NCBI Taxonomy database and references therein (Table S2). One type strain genome was chosen to represent each species.

### Ribosomal multilocus sequence typing (rMLST)

The rMLST scheme indexes the variation of the bacterial protein-encoding ribosomal genes [6]. There are 53 genes/loci defined in the scheme from across the 30S and 50S subunits of the ribosome (Table S3). Alleles are numbered in order of discovery within each locus, with each unique nucleotide sequence given a new allele number. Alleles are stored in the rMLST sequence definition library on the PubMLST website (https://pubmlst.org/species-id), e.g. BACT000035 allele 2393 (Fig. S2). The rMLST alleles for the 11839 *Klebsiella/Raoultella* genomes including the 17 type strains were identified and defined using a blast comparison with the existing rMLST allele library (4453 alleles).

A complete set of rMLST alleles for an isolate entry can be assigned as an rMLST profile (ribosomal sequence type, rST). For example, the unique combination of alleles observed for *Klebsiella aerogenes* type strain KCTC 2190 (id:23) is defined as rST:44287 and annotated as *K. aerogenes* (Fig. S3). In total, 11641 out of the 11839 *Klebsiella/Raoultella* entries were found to have a complete set of rMLST alleles (98.3%) and 2632 rSTs have been created and stored in the rMLST sequence definition library. The rMLST allele designations for each genome can be viewed on the isolate entry pages in the BIGSdb project database (Schemes and Loci section).

### rMLST nucleotide identity (rMLST-NI)

For any two genomes (a query and a reference), the DNA regions corresponding to rMLST loci can be identified, extracted and the number of identical bases between each pair of DNA sequences for the same rMLST locus calculated from a pairwise alignment. Summing the number of identical bases over all rMLST loci and dividing by the total number of aligned bases found in the reference genome across all the aligned rMLST loci gives a ‘local’ rMLST-based nucleotide identity. In practice, to speed up the comparison process, the DNA regions corresponding to rMLST loci within a set of reference genomes are defined in advance as rMLST alleles and each reference genome is summarized as an rMLST profile of allelic indices. The whole-genome sequence of the query is searched using blast against a DNA sequence library containing only the rMLST alleles defined by the set of reference profiles. The pairwise match information from genomic regions aligning to these rMLST alleles is collected, processed and the rMLST-NI values are calculated for all query/reference pairs simultaneously.

**Fig. 1.**
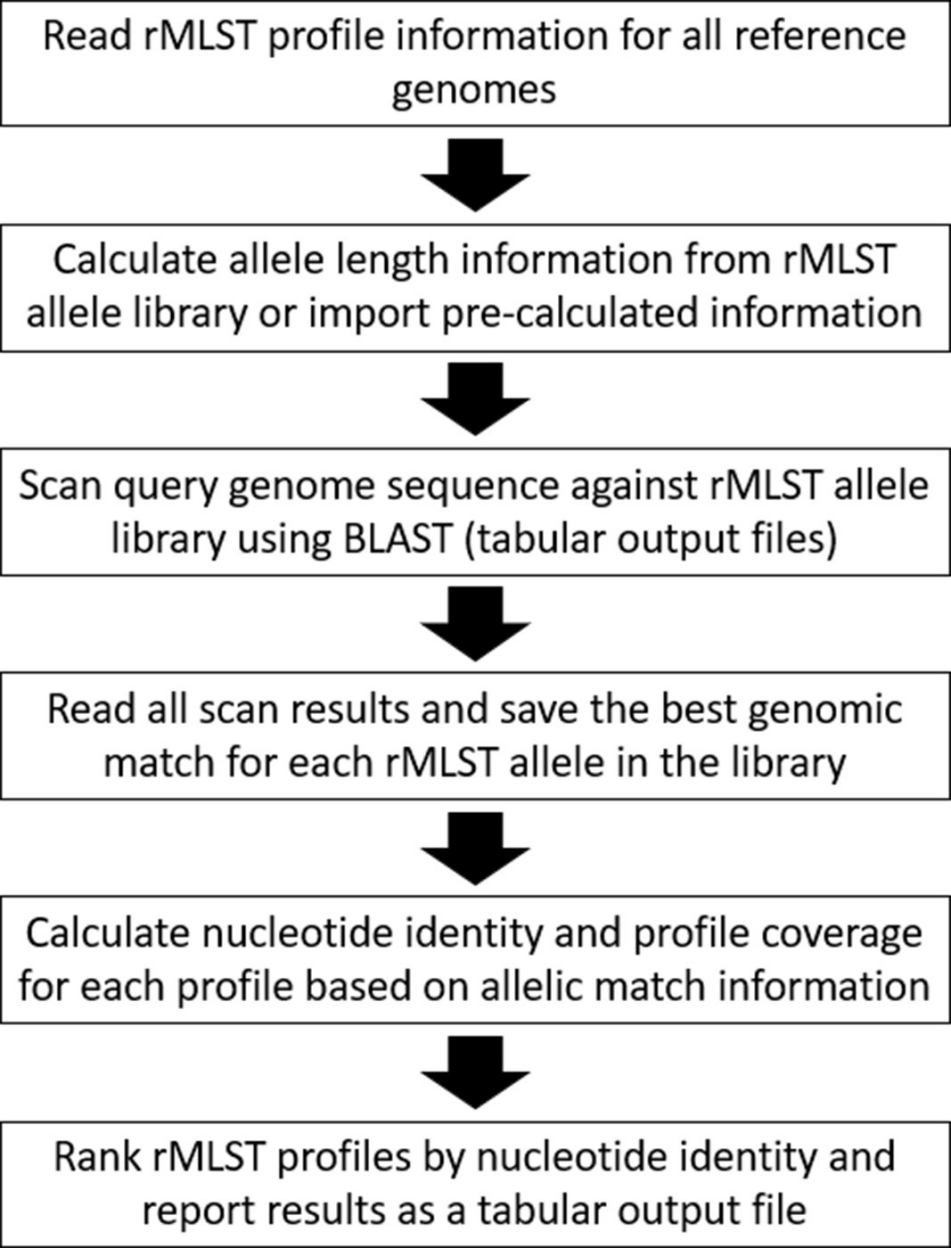
Flowchart describing the stages required to scan a query genome against a library of rMLST alleles and calculate the rMLST nucleotide identity (rMLST-NI) and profile coverage values. These stages are implemented in the MLSS.pl program (Multilocus Sequence Search).

**Fig. 2.**
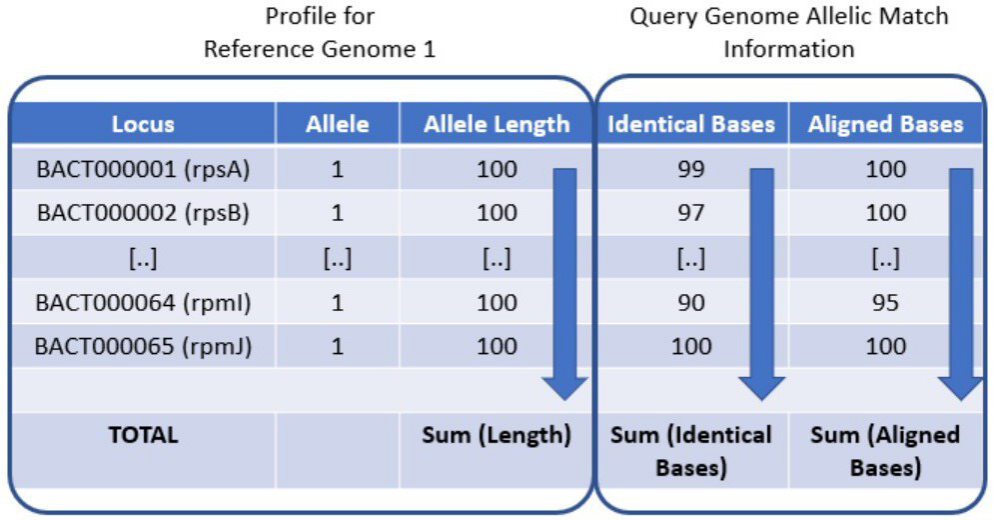
The rMLST nucleotide identity (rMLST-NI) for a query genome against each profile is calculated by extracting blast match information from each genome/allelic pairwise comparison (number of identical bases matched and the number of aligned bases). The rMLST-NI is defined as the total number of identical bases across all alleles in the profile divided by the sum of all aligned profile allele bases (expressed as a percentage). The profile coverage is similarly defined using the total number of aligned bases across all allelic matches. [··] indicates profile alleles and match information that have been removed. rMLST profiles typically contain allele indices for 53 loci. Allele indices and lengths shown are for illustrative purposes and do not represent the actual values.

The rMLST allele designations for the isolate records of 17 *Klebsiella/Raoultella* type strain entries were exported from the BIGSdb project database as a set of rMLST allelic profiles. In *Klebsiella/Raoultella* genomes, there are two rMLST genes that are typically found in multiple parts of the genome (BACT000060, *rpmE* and BACT000065, *rpmJ*) and, in accordance with rMLST profile convention, these were excluded from the nucleotide identity calculation. These positions in the profile table were annotated with the letter N. The corresponding DNA sequences of 618 rMLST alleles from the remaining 51 loci were extracted from the rMLST sequence definition library and combined into one FASTA sequence file suitable for searching with blastn. A Perl program (MLSS.pl v1.0.0, Multilocus Sequence Search) was written to combine the tasks of running blastn (version 2.12.0+, June 2021) against the allelic sequence library (word size 30), parsing the tabular blast output and calculating the nucleotide identity for each representative rMLST profile (Fig. 1). The best sequence match for each rMLST allele was defined as the match with the highest blast score. A local rMLST-NI for each profile in the library was calculated by summing the number of identical bases across the matches to alleles for that profile and dividing by the total number of aligned profile bases (expressed as a percentage). The percentage overlap was similarly calculated from the total number of aligned bases divided by the total length of all the alleles in the profile (Fig. 2). A local identity metric was chosen to prevent relatively small amounts of missing genomic DNA having a large impact on the final sequence identity value and its subsequent interpretation.

### Whole-genome average nucleotide identity using OrthoANIu software

The genome sequences of each of the 17 *Klebsiella/Raoultella* type strain isolates were exported from the BIGSdb project database and stored as individual files. The OrthoANIu program was used to calculate a pairwise average nucleotide identity for two genome sequences [20, 21]. The algorithm chops the genome sequences into 1020 bp long fragments and identifies which DNA fragments to compare based on reciprocal matches (orthologous relationships). In OrthoANIu, the USEARCH algorithm is used rather than blastn to decrease computational time. The java program OAU.jar (v1.2, https://www.ezbiocloud.net/tools/orthoaniu) and USEARCH binary file (‘usearch11.0.667_i86linux32’, http://www.drive5.com/usearch/download.html) were used in this study.

### Whole-genome average nucleotide identity using FastANI software

The FastANI program (v1.33, https://github.com/ParBLiSS/FastANI) was used to calculate an estimated pairwise average nucleotide identity between each query genome sequence and the 17 *Klebsiella/Raoultella* type strain genomes [3]. FastANI uses the Mashmap algorithm to map 3 Kbp non-overlapping fragments of a query genome sequence onto a reference genome [25]. The mapped query fragments are filtered to remove mappings to the same or nearby reference positions and the average nucleotide identity is estimated from calculating the shared *k*-mers within each of the remaining mapped regions (default *k*-mer size 16). This is an alignment-free approach and results in significantly reduced computational time requirements compared with alignment-based methods that use blastn or USEARCH [3].

### Kleborate species scan

The MinHash sketch library downloaded with the Kleborate software (v0.4.0-beta, https://github.com/katholt/Kleborate) contains 1034 entries for *Klebsiella/Raoultella* genomes out of a total of 2620 library entries. Examination of the Kleborate library revealed parameters set to *k*-mer sizes of 21 and 1000 *k*-mers per sketch. The mash software [26] is used to search a query genome against the sketch library by first converting the query genome into a sketch using the MinHash algorithm and then comparing the number of *k*-mers in common with each sketch library entry. Kleborate parses the mash output to identify the library entry sharing the most *k*-mers with the query genome and describes the match as either ‘strong’ or ‘weak’ based on the observed distance metric generated from the pairwise comparison. Strong matches are less than or equal to 0.02 (approximately 98 % identity) and weak matches are more than 0.02 and less than or equal to 0.04 (approximately between 98 and 96% identity). The Kleborate species library contains representatives of all 17 *Klebsiella/Raoultella* species described in this study.

### Traffic light scheme for assessing similarity significance

The complete scan results from the dataset of 11733 entries with species annotations that were consistent with the NCBI Assembly annotation were combined with the complete scan results from the 17 type strains (all 17 comparisons per query). A total of 199 750 pairwise comparisons were analysed for each of the three methods that yield a nucleotide identity value in the results (OrthoANIu, FastANI and rMLST-NI). The species annotations of the query and library genomes were added to the scan results and the species of each pairwise comparison was compared and designated as ‘true’ (species match) or ‘false’ (species mismatch). The scan results of all the 11 750 matches to each library genome (type strain) were sorted by sequence identity (largest first) and the table descended until the last ‘true’ scan result pair was identified before ‘false’ pairs were observed. The sequence identity of this scan result was assigned as threshold A for each library genome. The sequence identity of the highest identity ‘false’ pair was assigned as threshold B for each library genome.

A ‘traffic light’ colour scheme was implemented to simplify assessing the significance of a nucleotide identity value. In this scheme, the sequence identity range equal to or greater than threshold A were designated as ‘green’ zone matches. Sequence identities found to be equal or below threshold B were designated as ‘red’ zone matches. Sequence identities lower than threshold A and higher than threshold B were assigned ‘amber’ zone matches. A query genome that matched to a type strain library genome with a nucleotide identity value in the green zone could be predicted to have the species of the library genome, given that there have been no observations of mismatched species annotation pairs within this sequence identity range in the dataset of 11 750 genomes. The nucleotide identity thresholds for the 17 *Klebsiella/Raoultella* type strains were calculated for OrthoANIu and FastANI (Table S4), and the rMLST-NI method (Table S5).

### DNA contamination detection using rMLST alleles

The genome sequences in the 11 839 dataset were scanned against the rMLST allele sequence database (https://pubmlst.org/species-id) using blastn and the positions of all exactly matching rMLST alleles were recorded for each genome. The associated allele numbers of each match were automatically recorded in the *Klebsiella/Raoultella* project database. The rMLST allele designations are available to view within each isolate record page under the ‘Schemes and Loci’ section.

BIGSdb software dynamically calculates the observed species annotations of each rMLST allele based on allele designation information found within the PubMLST Multi-species database (https://pubmlst.org/species-id). rMLST alleles found to be present in the 11 839 dataset that have been identified as present in exclusively non-*Klebsiella/Raoultella* genomes in the Multi-species database were noted (Supplementary Material: Excel spreadsheet, AlleleResults tab). Forty-eight genomes contained at least one rMLST allele matching this description, e.g. id: 4663 (SL34, GCA_003363575.1, *Klebsiella pneumoniae *) contained the BACT000010 (*rpsJ*) allele 128 that is found in *Acinetobacter baumannii* genomes. The BIGSdb *Klebsiella/Raoultella* project database has a field in the isolate table named ‘contaminated’, which was populated by ‘suspected’ for genomes where at least one exclusively non-*Klebsiella/Raoultella* rMLST allele was detected along with a short description in the comments section.

## Results

### Calculation of program execution times

All programs were run on a Dell PowerEdge R815 Server with 512 Gb of RAM and 64 CPU cores, using the Ubuntu operating system (release 18.04). The genome sequences of the 17 type strain isolates were scanned against each library and the program log files examined to determine the time taken to complete the jobs (Table S6). OrthoANIu could be run on multiple processing cores (modified with -n flag) and program execution times were calculated for 1, 10, 20 CPU. Based on the execution time for 1 CPU core, the rMLST-NI scan took 52 s (using MLSS.pl v1.0.0 and blast v2.12.0+), the Kleborate species scan took 179 s, FastANI took 288 s, and OrthoANIu took 84 651 s (23 h 30 m 51 s). Based on the timings for 1 CPU, rMLST-NI is around 3.5 times faster than the Kleborate species scan, 4.5 times faster than FastANI and approximately 1600 times faster than OrthoANIu.

### Comparison of species annotations and species predictions

**Fig. 3.**
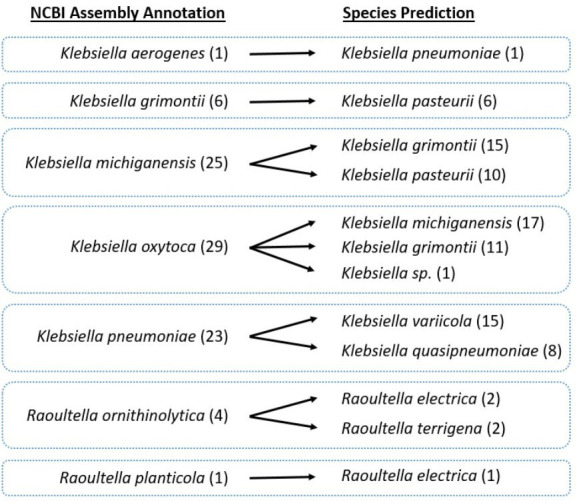
Summary of 89 inconsistent species annotations where the NCBI Assembly database has a different species than the consensus result obtained from the four automated approaches used in this study (OrthoANIu, FastANI, rMLST-NI and Kleborate). The left-hand side represents the original species annotations from NCBI Assembly and the right-hand side represents the predicted species.

The dataset of 11822 *Klebsiella/Raoultella* non-type strain isolates were analysed using the three automated species identification methods (OrthoANIu, rMLST-NI and Kleborate species scan). All isolates were found to have consistent species prediction results across all three methods according to the species of the highest identity type strain (OrthoANIu, rMLST-NI) or reported species (Kleborate) (Supplementary Material: Excel spreadsheet, ScanResults tab). Comparison with the species annotation reported in the NCBI Assembly record on 5 March 2021, identified 11733 records where the predicted species agreed with the source database and 89 records where the species identified was different. Where there was an annotation discrepancy (Fig. 3), the predicted species was generally a more recent species definition than the source database entry, e.g. there were 25 isolates annotated as *K. michiganensis* (type strain published in 2013 [27]) that are predicted to be either *K. grimontii* (*n*=15, published in 2018 [28]) or *K. pasteurii* (*n*=10, published in 2019 [23]).

**Fig. 4.**
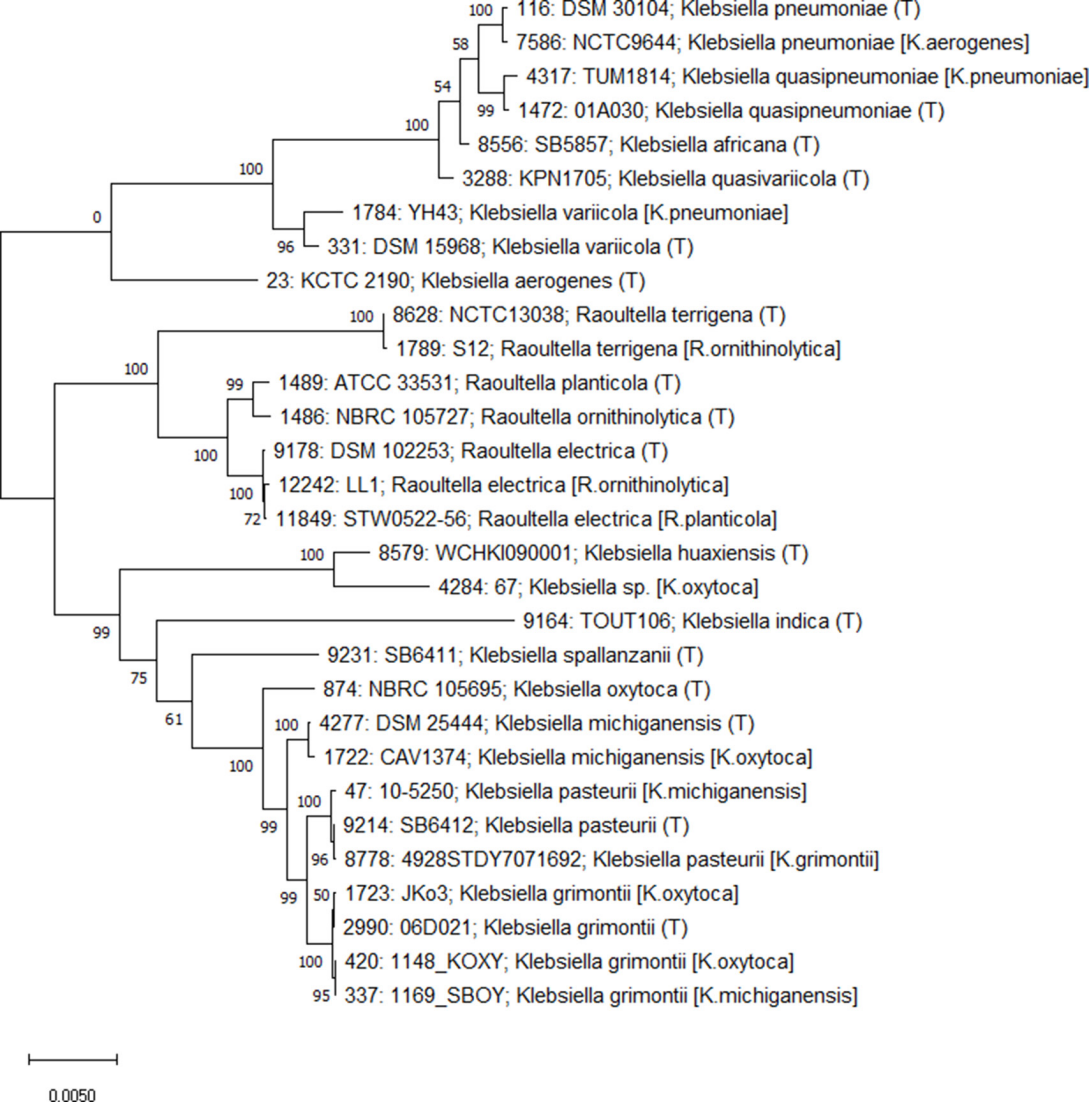
Phylogenetic tree of 30 *Klebsiella/Raoultella* isolates including 17 type strains and 13 non-type strain isolates where the NCBI Assembly species annotation was not consistent with the automated species identification scan results. Each isolate is annotated with (1) database identifier, (2) isolate name, (3) species (T indicates type strain), (4) species in source database (shown in square brackets if different). This tree was constructed in mega X (www.megasoftware.net) using the maximum-likelihood method and Tamura–Nei model (1000 replicates) from a multiple sequence alignment of the concatenated alleles of 51 non-paralogous rMLST loci generated by BIGSdb.

Examination of the OrthoANIu results for these 89 isolates was conducted whereby the ANI for the closest type strain genome was compared with the ANI for the type strain based on the species annotation at NCBI Assembly from 5 March 2021 (Table S7). For example, isolate 1169_SBOY (GCA_001060405.1, id:337) is recorded as *K. michiganensis* in the source database (93.8%) but the closest type strain genome is *K. grimontii* (99.2 %). The ANI value for the closest type strain was typically more than 96 %, consistent with previous studies of determining ANI values for species demarcation [1], whereas the ANI value for the source database species annotation was typically less than 96 %, which was supported by phylogenetic analysis (Fig. 4) using the 17 type strains and 13 exemplar NCBI Assembly entries with annotation discrepancies (Table 2). The non-type strain mis-annotated entries cluster with the nearest type strain identified in this study rather than the type strain based on the source database annotation. The BIGSdb software allows users to rapidly generate neighbour-joining phylogenetic trees using clustal W [29] (v2.1) and upload them to the Interactive Tree of Life (iTOL) [30] tree visualization website (Fig. S4). This approach can provide a rapid visualization of the position of a query genome on a phylogenetic tree of type strain genomes prior to performing more time-consuming analyses.

**Table 2.** Summary of OrthoANIu results for 13 NCBI Assembly entries where the NCBI Assembly database species annotation does not correspond with the closest *Klebsiella/Raoultella* type strain using ANI as the genomic comparison metric. The ANI value for the closest type strain species and the source database species are included

Isolate Id	Isolate name	NCBI Assembly identifier (GenBank)	Species of best OrthoANIu match (average nucleotide identity)	Species in source database (average nucleotide identity)
7586	NCTC9644	GCA_900636315.1	* K. pneumoniae* (99.1%)	* K. aerogenes* (84.9%)
8778	4928STDY7071692	GCA_902163325.1	* K. pasteurii* (99.4%)	* K. grimontii* (96.0%)
337	1169_SBOY	GCA_001060405.1	* K. grimontii* (99.2%)	* K. michiganensis* (93.8%)
47	10–5250	GCA_000247915.1	* K. pasteurii* (99.4%)	* K. michiganensis* (93.9%)
420	1148_KOXY	GCA_001052235.1	* K. grimontii* (99.2%)	* K. oxytoca* (91.4%)
1723	JKo3	GCA_001548355.1	* K. grimontii* (99.2%)	* K. oxytoca* (91.4%)
4284	67	GCA_003201885.1	* K. huaxiensis* (95.3%)	* K. oxytoca* (87.2%)
1722	CAV1374	GCA_001022195.1	* K. michiganensis* (98.9%)	* K. oxytoca* (92.3%)
4317	TUM1814	GCA_003176335.1	* K. quasipneumoniae* (96.6%)	* K. pneumoniae* (93.8%)
1784	YH43	GCA_001548315.1	* K. variicola* (97.0%)	* K. pneumoniae* (94.2%)
12 242	LL1	GCA_014764465.1	* R. electrica* (98.8%)	* R. ornithinolytica* (94.1%)
1789	S12	GCA_000829965.1	* R. terrigena* (99.1%)	* R. ornithinolytica* (85.4%)
11 849	STW0522-56	GCA_015135635.1	* R. electrica* (99.0%)	* R. planticola* (93.5%)

### Whole genome ANI results (OrthoANIu and FastANI)

The OrthoANIu scan results for the 11733 genome records with species predictions consistent with NCBI Assembly annotations were analysed to define species-specific ANI thresholds for annotation inheritance. The highest observed match to a *Klebsiella/Raoultella* type strain with a different species annotation was typically 96 % or lower for each of the 17 type strains (Table S4). The highest ANI match for each of the 11733 query isolates was a type strain genome with a nucleotide identity in the green zone (Table 3). Of the 89 genomes with a prediction that disagreed with the NCBI Assembly annotation, there were 83 cases where the pairwise identity of the closest type strain was found to be in the green zone confirming the predicted species. In five of the remaining six cases the ANI value of the closest type strain match was outside the green zone but was found to be above 98.8 % identity, therefore sufficiently high to be considered to be correct predictions (ids: 441, 8616, 11849, 11925, 12242). The species field of these 88 records in the *Klebsiella* project database were modified to reflect the predicted species. The original species from the NCBI Assembly database was added to the field ‘species in source database’. Isolate 67 (GCA_003201885.1, id:4284) was found to have 95.3 % identity with the type strain for *K. huaxiensis* (compared with 87.2 % for the NCBI annotation *K. oxytoca *) and was therefore assigned as *Klebsiella* sp. in the project database as 95.3 % was not sufficiently similar to merit the species assignment. A summary of the total number of members of each species across the 11839 genomes in the *Klebsiella* project database based on the results of this study is described in Table 4.

**Table 3.** Summary of species identification results for the four methods against two genomic datasets. The dataset of 11733 genomes included entries where the species prediction agreed with the NCBI Assembly annotation and the dataset of 89 genomes where the species prediction disagreed. The species of the top matching type strain was the same for all four methods across the 11822 entries

Dataset	Method	Green-zone inheritance confidence	Amber-zone inheritance confidence	Red-zone inheritance confidence
11 733 genomes	OrthoANIu	11 733 (100 %)	0 (0 %)	0 (0 %)
11 733 genomes	FastANI	11 733 (100 %)	0 (0 %)	0 (0 %)
11 733 genomes	rMLST-NI	11 730 (99.97 %)	0 (0 %)	3 (0.03 %)
11 733 genomes	Kleborate	11 705 (99.76 %)	28 (0.24 %)	0 (0 %)
89 genomes	OrthoANIu	83 (93.26 %)	6 (6.74 %)	0 (0 %)
89 genomes	FastANI	83 (93.26 %)	6 (6.74 %)	0 (0 %)
89 genomes	rMLST-NI	84 (94.38 %)	5 (5.62 %)	0 (0 %)
89 genomes	Kleborate	89 (100 %)	0 (0 %)	0 (0 %)

**Table 4.** Number of genomes from the *Klebsiella* and *Raoultella* genera in the *Klebsiella* project database (as of 28 April 2021)

Species	No. of genomes with consistent species annotations (no. of type strains)	No. of genomes with inconsistent species annotations (predicted species shown)
* Klebsiella aerogenes *	291 (1)	0
* Klebsiella africana *	1 (1)	0
* Klebsiella grimontii *	96 (1)	26
* Klebsiella huaxiensis *	2 (1)	0
* Klebsiella indica *	0 (1)	0
* Klebsiella michiganensis *	223 (1)	17
* Klebsiella oxytoca *	161 (1)	0
* Klebsiella pasteurii *	12 (1)	16
* Klebsiella pneumoniae *	9765 (1)	1
* Klebsiella quasipneumoniae *	586 (1)	8
*Klebsiella quasivariicola*	13 (1)	0
*Klebsiella sp*.	0	1
* Klebsiella spallanzanii *	3 (1)	0
* Klebsiella variicola *	430 (1)	15
* Raoultella electrica *	0 (1)	3
* Raoultella ornithinolytica *	79 (1)	0
* Raoultella planticola *	36 (1)	0
* Raoultella terrigena *	35 (1)	2
**TOTAL**	**11 733 (17**)	**89**

The species prediction results based on alignment-free FastANI comparisons were congruent with the alignment-based OrthoANIu results. All 11733 query genomes with a species prediction that matched the NCBI Assembly annotation yielded a nucleotide identity in the green zone (Table 3). For the 89 genomes where the predicted species disagreed with the NCBI Assembly annotation, the predicted species was the same as that identified as the highest match by OrthoANIu. Similarly, five genomes gave amber zone results (ids: 441, 8616, 11849, 11925, 12242) with an ANI value of greater than 98.6 % and id: 4284 resulted in a 95.6 % amber zone match to the *Klebsiella huaxiensis* type strain.

The pairwise ANI values from the OrthoANIu and FastANI algorithms were examined based on the comparisons between the entire 11839 genomic dataset and the 17 genome type strain library (Fig. S5). These results show that the alignment-free FastANI algorithm is a reliable estimator of ANI values between pairs of *Klebsiella/Raoultella* genomes (R^2^ value is 0.999).

### rMLST nucleotide identity (rMLST-NI)

The rMLST-NI method correctly identified the species of the NCBI Assembly record for the 11733 query genomes as the closest *Klebsiella/Raoultella* type strain. This was consistent with the results from OrthoANIu and the Kleborate species scan. The observed pairwise nucleotide identities for each type strain species were compiled to define the identity thresholds required for the traffic light scheme as described previously for the OrthoANIu results. The thresholds were calculated (Table S5) and the results of the scan results of the 11733 isolate dataset were re-analysed to assign each nucleotide identity a green/amber/red level of significance. For 11730/11733 (99.97 %) of the species predictions, there was a rMLST nucleotide identity in the green zone indicating that the species of the matching profile could be reliably inherited. Three predictions were in the red zone indicating that the identity was below that observed for the highest profile match with a different species annotation (Table 3). In two cases there was incomplete genomic data combined with contaminating DNA from a non-*Klebsiella/Raoultella* species (ids: 4528 and 8691). In the third case (id: 2293), one poorly assembled locus reduced the identity into the red zone (BACT000043, *rplN*). Of the 89 inconsistently annotated genomes, 84 were green zone matches and five were in the amber zone (ids: 4284, 8616, 11849, 11925, 12242). As described above for OrthoANIu, id: 4284 (match to type strain for *K. huaxiensis *) was identified as not having sufficient similarity to merit the species assignment. Three entries were identified as *R. electrica *. (Supplementary Material, Excel spreadsheet). For the rMLST-NI searches conducted in this study, the threshold A for *R. electrica* was set to 100 % as there were no other members of this species available for observing sequence variation. In future analyses, a modified threshold allowing for a small number of nucleotide differences (e.g. 10) would be more appropriate for cases where the training dataset only contains one member of a particular species. The last remaining amber zone result (id: 11925) correctly matched to the *K. pasteurii* type strain but with slightly more ribosomal gene diversity than observed in the 12 *K *. *pasteurii* genomes in the training dataset leading to an identity value just below threshold A. As more genomic data becomes available for less well-sampled members of the *Klebsiella/Raoultella* genera the rMLST-NI thresholds can be adjusted accordingly.

### Kleborate species scan

The Kleborate species scan results describe the species prediction as either strong or weak depending on the value of the mash distance (not reported in the Kleborate output file). Overall, 11705 of the 11733 query genomes were assigned as strong matches (distance ≤0.02) and 28 were weak matches (distance ≤0.04 and>0.02). All 11733 predicted species agreed with the NCBI database species annotation. All 89 of the inconsistently annotated set of genomes were assigned to a species as a strong match (Table 3).

### rMLST-NI webserver

**Fig. 5.**
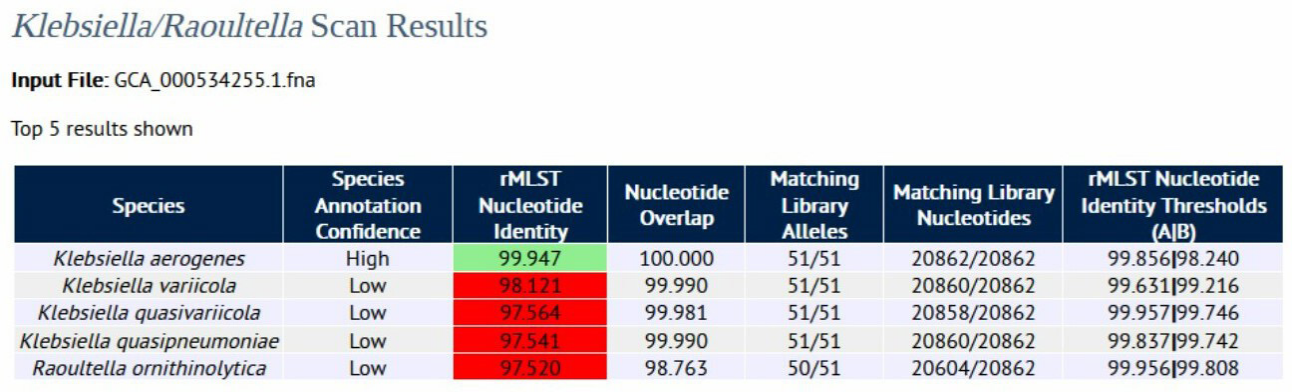
rMLST nucleotide identity webserver results for scanning UCI 27, (NCBI Assembly entry GCA_000534255.1, *Klebsiella aerogenes *). The top five species are displayed in the results table and are colour coded according to the traffic light system of thresholds observed from the analysis of scan results from the 11750 dataset.

A webserver that scans a genome sequence against the rMLST alleles of the library of 17 *Klebsiella/Raoultella* type strain isolates to calculate the rMLST nucleotide identity of each pairwise comparison has been developed (https://pubmlst.org/species-id/klebsiella). An example of a genomic scan using UCI 27 (GCA_000534255.1, id: 192, *Klebsiella aerogenes *) is shown in Fig. 5. The top five results are shown ranked by rMLST nucleotide identity so that the most similar isolate in the library is ranked first. The pairwise comparison between the query genome and type strain profile entry id: 55531 (* Klebsiella aerogenes *) has an rMLST-NI value of 99.947 % and a nucleotide overlap of 100 %. This rMLST-NI value falls into the green zone of observed identities, whereas the other four type strain matches fall in the red zone (* K. variicola *, *K. quasivariicola*, *K. quasipneumoniae* and *R.ornithinolytica*). The query genome can be reliably annotated as *K. aerogenes* based on these results.

## Discussion

As the diversity of bacterial species is increasingly explored through WGS, integrated with other technologies and data sources, there is a growing need to actively monitor and update the taxonomic annotations in publicly available databases. This is especially the case when molecular approaches are used for clinical purposes, as clinical management can be reliant on taxonomic designations. It is therefore also important that there is transparency about how species annotations are derived, when they were last updated, and what reference datasets were used in their derivation. As the capacity to generate large quantities of genomic data increases rapidly, so must the development of databases and tools capable of managing taxonomic annotation changes. This is, however, both computationally intensive and time-consuming. We therefore developed an efficient method to calculate and report the rMLST nucleotide identity (rMLST-NI) between a query genome and a library of type strain genomes based on their ribosomal protein-encoding genes. This approach can be applied to any bacterial species with defined type strains. The WGS of each query genome was searched against a small DNA library of pre-calculated rMLST allelic variants facilitating the rapid calculation of a set of pairwise rMLST nucleotide identities.

The method has been applied to a large dataset of publicly available *Klebsiella/Raoultella* genomes. The rMLST-NI method gave the same species prediction results as three whole-genome-based methods, OrthoANIu, FastANI and Kleborate species scan. The rMLST-NI method was approximately 3.5 times faster than the Kleborate species scan, 4.5 times faster than FastANI and 1600 times faster than OrthoANIu, while still dependent only upon a DNA-based library derived from type strain genomes. The wgANI method was considered to be the gold standard for computational species identification at the time of writing [1], so it was reassuring that rMLST-NI performed with comparable accuracy and improved speed. FastANI was also shown to provide the same species identification results as OrthoANIu but was 360 times faster in our tests.

For the *Klebsiella/Raoultella* genera, the Kleborate application has been developed by the research community due to the major biomedical interest in these organisms [22]. Hence, it was possible to compare rMLST-NI to an application that has been developed specifically to address *Klebsiella/Raoultella* species nuances. This demonstrated that rMLST-NI was comparable in accuracy and slightly faster. The need to consistently and continuously update genomic collections as new *Klebsiella/Raoultella* species are discovered is clear and by using the rMLST-NI method, the type strain DNA library of rMLST allelic variants can be easily updated. The *Klebsiella/Raoultella* dataset of 11839 genomes could be re-analysed in approximately 10 h (using MLSS.pl and 1 CPU core) to assess the impact of new species definitions on existing annotations and redefine the species-specific nucleotide identity thresholds. The Kleborate species scan method is based on the species annotations of more than 1000 *Klebsiella/Raoultella* genomes and relies on these annotations being accurate across the entire library. The mash distance metric was shown to be a robust measure of the significance of the top matching species. The Kleborate approach would therefore also be an efficient method to re-analyse many thousands of genomes provided that the Kleborate library could be demonstrated to contain the latest *Klebsiella/Raoultella* species definitions (updated by the Kleborate curators) and all library members were correctly annotated.

Species-specific nucleotide identity thresholds were calculated based on the observations from 11750 species validated NCBI Assembly entries (11733 plus 17 type strains), and a traffic light system implemented to indicate whether the highest identity species prediction could be reliably assigned to the query genome. For rMLST-NI, this system was sufficiently robust to assign the species annotation automatically for 99.97 % of the subset of predictions agreeing with NCBI Assembly annotations. In the context of this study, the query genomes were already known to be species within the *Klebsiella/Raoultella* genus and the four species identification methods were used to distinguish among the 17 possible species; however, if the genus/species of the query genome were unknown, a red zone match using rMLST-NI and this *Klebsiella/Raoultella* constrained library would not be sufficient evidence to confirm the species of the query genome. To widen the scope of the rMLST-NI method, therefore, type strains from a broader range of bacterial species need to be identified and curated, for situations where the species of the query genome is less constrained. This represents a limitation of current practice within genomic collections, where type strains are (i) not always readily available for all new species in a timely fashion and (ii) not consistently curated. This is a challenge that the microbial genomics and systematics communities need to embrace, and it is incumbent upon those identifying and submitting new species for recognition, to ensure that the existing genomic repositories, on which so many are dependent, remain fit for purpose. A further limitation of the current rMLST-NI method is the derivation of evidence-based species-specific nucleotide identity thresholds for a species with only one member in the dataset. This is not typically a problem for pathogenic bacterial species that have been sequenced many times, however, for low sampled species the use of conservative generic thresholds may be appropriate until more data are available to refine the threshold values. Increasingly, metagenomic sequencing of bacterial communities is being performed but as yet rMLST-NI has not been tested or validated on low quality or metagenomic data where there may be insufficient ribosomal gene coverage compared to WGS to yield a result.

This study identified 89 NCBI Assembly database entries with incorrect species annotations for *Klebsiella/Raoultella* genomes. These were subsequently corrected in the BIGSdb project database through a process involving human input, demonstrating the practical difficulty in continuously maintaining accurate species annotations across large multi-species genomic repositories in the face of changing species definitions. Automating such methods to periodically check species annotations using fast, generic methods will reduce this workload and improve accuracy in the long term. The use of these genomic repositories by third party users such as researchers and clinicians mean that there is an urgent and ongoing need for them to remain accurate. Diagnostic tools that utilize DNA-based technologies such as organism-specific quantitative PCR (qPCR) or broad range 16S PCR can be developed to identify pathogenic species of bacteria [31]. Both these methods require the use of large genomic databases for the development of such assays (qPCR) and the subsequent analysis (16S PCR). The development and assessment of new vaccines, antimicrobial drugs or phage therapies requires an understanding of species-specific genetic elements and therefore that species annotations are accurately maintained.

Here we demonstrate a method to rapidly validate the species designation of *Klebsiella/Raoultella* genomes using 17 type strain genomes as the reference point. We applied the method to more than 11000 *Klebsiella/Raoultella* genomes and identified 89 incorrect annotations. The rMLST nucleotide identity (rMLST-NI) method uses the DNA sequences of genes that encode ribosomal proteins and, as these genes are present in all bacteria, this approach can be potentially developed to validate the species annotations of many other bacterial pathogens. This broad applicability in addition to the accuracy of the rMLST-NI method, combined with modest computational requirements makes this a practical tool in helping to resolve the bacterial species annotation problems that high-throughput DNA sequencing generates.

## Supplementary Data

Supplementary material 1

Supplementary material 2
