# Supplementary material for: Ribosomal MLST nucleotide identity (rMLST-NI), a rapid bacterial species identification method: application to Klebsiella and Raoultella genomic species validation
Source: Microb Genom. 2022 Sep 13;8(9):mgen000849. doi: 10.1099/mgen.0.000849 (PMC9676034; doi:10.1099/mgen.0.000849)
Supplement: Supplementary material 1 [file mgen-8-849-s001.pdf]

Assessment of species identification methods for the large-scale validation of *Klebsiella* and *Raoultella* species annotations in bacterial genome databases

Supplementary Data

Tables and Figures

Database ID	Isolate Name	NCBI GenBank assembly accession	Species in Publication	Previous species annotation	NCBI BioSample accession(s)	PubMed ID
8556	SB5857	GCA_900978845.1	*Klebsiella africana*	*Klebsiella quasipneumoniae*	ERS2787533; SAMEA4969318	30817987
8663	SB3355	GCA_901563825.1	*Klebsiella pasteurii*	*Klebsiella grimontii*	ERS3414262; SAMEA5610071	31708881
8666	SB3356	GCA_901563875.1	*Klebsiella spallanzanii*	*Klebsiella michiganensis*	ERS3414261; SAMEA5610070	31708881
9187	SB6413	GCA_902158715.1	*Klebsiella pasteurii*	*Klebsiella michiganensis*	ERS3414268; SAMEA5610077	31708881
9211	SB6407	GCA_902158585.1	*Klebsiella pasteurii*	*Klebsiella grimontii*	ERS3414263; SAMEA5610072	31708881
9212	SB6409	GCA_902158575.1	*Klebsiella pasteurii*	*Klebsiella grimontii*	ERS3414267; SAMEA5610076	31708881
9213	SB6410	GCA_902158545.1	*Klebsiella pasteurii*	*Klebsiella grimontii*	ERS3414264; SAMEA5610073	31708881
9214	SB6412	GCA_902158725.1	*Klebsiella pasteurii*	*Klebsiella grimontii*	ERS3414265; SAMEA5610074	31708881
9215	SB6414	GCA_902158655.1	*Klebsiella pasteurii*	*Klebsiella grimontii*	ERS3414269; SAMEA5610078	31708881
9216	SB6415	GCA_902158635.1	*Klebsiella pasteurii*	*Klebsiella grimontii*	ERS3414270; SAMEA5610079	31708881
9217	SB6416	GCA_902158665.1	*Klebsiella pasteurii*	*Klebsiella grimontii*	ERS3414271; SAMEA5610080	31708881
9218	SB6417	GCA_902158695.1	*Klebsiella pasteurii*	*Klebsiella grimontii*	ERS3414272; SAMEA5610081	31708881
9219	SB6420	GCA_902158645.1	*Klebsiella pasteurii*	*Klebsiella grimontii*	ERS3414273; SAMEA5610082	31708881
9220	SB6423	GCA_902158705.1	*Klebsiella pasteurii*	*Klebsiella grimontii*	ERS3414274; SAMEA5610083	31708881
9221	SB6424	GCA_902158685.1	*Klebsiella pasteurii*	*Klebsiella grimontii*	ERS3414266; SAMEA5610075	31708881
9230	SB6408	GCA_902158595.1	*Klebsiella spallanzanii*	*Klebsiella michiganensis*	ERS3414258; SAMEA5610067	31708881
9231	SB6411	GCA_902158555.1	*Klebsiella spallanzanii*	*Klebsiella michiganensis*	ERS3414259; SAMEA5610068	31708881
9232	SB6419	GCA_902158565.1	*Klebsiella spallanzanii*	*Klebsiella michiganensis*	ERS3414260; SAMEA5610069	31708881

Supplementary Data Table 1: Species annotations for 18 genome records available at the NCBI Assembly database on 5th March 2021 were updated based on the information found in two publications (PMID:31708881, PMID:30817987) and communication with the publication authors (Carla Rodrigues, Institute Pasteur).

Database ID	Isolate Name	Species	NCBI GenBank Assembly Accession	NCBI BioSample Accession	Country	Year of Isolation
23	KCTC 2190	*Klebsiella aerogenes*	GCA_000215745.1	SAMN02603581	n/a	n/a
8556	SB5857	*Klebsiella africana*	GCA_900978845.1	SAMEA4969318	Senegal	2016
2990	06D021	*Klebsiella grimontii*	GCA_900200035.1	SAMEA104188450	France	1997
8579	WCHKl090001	*Klebsiella huaxiensis*	GCA_003261575.2	SAMN08861555	China	2017
9164	TOUT106	*Klebsiella indica*	GCA_005860775.1	SAMN11776007	India	2014
4277	DSM 25444	*Klebsiella michiganensis*	GCA_002925905.1	SAMN07187491	USA	2010
874	NBRC 105695	*Klebsiella oxytoca*	GCA_001598695.1	SAMD00046899	USA	n/a
9214	SB6412	*Klebsiella pasteurii*	GCA_902158725.1	SAMEA5610074	Italy	2017
116	DSM 30104	*Klebsiella pneumoniae*	GCA_000281755.1	SAMN02470063	n/a	n/a
1472	01A030	*Klebsiella quasipneumoniae*	GCA_000751755.1	SAMEA2471851	Austria	1997
3288	KPN1705	*Klebsiella quasivariicola*	GCA_002269255.1	SAMN06438648	USA	2014
9231	SB6411	*Klebsiella spallanzanii*	GCA_902158555.1	SAMEA5610068	Italy	2017
331	DSM 15968	*Klebsiella variicola*	GCA_000828055.2	SAMN01174581	Mexico	n/a
9178	DSM 102253	*Raoultella electrica*	GCA_006711645.1	SAMN12125603	Japan	2013
1486	NBRC 105727	*Raoultella ornithinolytica*	GCA_001598295.1	SAMD00046738	Japan	n/a
1489	ATCC 33531	*Raoultella planticola*	GCA_000735435.1	SAMN02743268	n/a	n/a
8628	NCTC13038	*Raoultella terrigena*	GCA_900706855.1	SAMEA2580322	n/a	n/a

Supplementary Data Table 2: 17 type strain isolates for the *Klebsiella/Raoultella* genera used in this study. The table contains the numeric database identifier in the Klebsiella project database (https://pubmlst.org/projects/klebsiella), the isolate name, species annotation, NCBI Assembly accession, NCBI BioSample accession, country of origin and year of isolation. n/a indicates where a country or year of isolation is not available.

rMLST Locus Identifier	Gene Name	Protein Product
BACT000001	*rpsA*	30S ribosomal protein S1
BACT000002	*rpsB*	30S ribosomal protein S2
BACT000003	*rpsC*	30S ribosomal protein S3
BACT000004	*rpsD*	30S ribosomal protein S4
BACT000005	*rpsE*	30S ribosomal protein S5
BACT000006	*rpsF*	30S ribosomal protein S6
BACT000007	*rpsG*	30S ribosomal protein S7
BACT000008	*rpsH*	30S ribosomal protein S8
BACT000009	*rpsI*	30S ribosomal protein S9
BACT000010	*rpsJ*	30S ribosomal protein S10
BACT000011	*rpsK*	30S ribosomal protein S11
BACT000012	*rpsL*	30S ribosomal protein S12
BACT000013	*rpsM*	30S ribosomal protein S13
BACT000014	*rpsN*	30S ribosomal protein S14
BACT000015	*rpsO*	30S ribosomal protein S15
BACT000016	*rpsP*	30S ribosomal protein S16
BACT000017	*rpsQ*	30S ribosomal protein S17
BACT000018	*rpsR*	30S ribosomal protein S18
BACT000019	*rpsS*	30S ribosomal protein S19
BACT000020	*rpsT*	30S ribosomal protein S20
BACT000021	*rpsU*	30S ribosomal protein S21
BACT000030	*rplA*	50S ribosomal protein L1
BACT000031	*rplB*	50S ribosomal protein L2
BACT000032	*rplC*	50S ribosomal protein L3
BACT000033	*rplD*	50S ribosomal protein L4
BACT000034	*rplE*	50S ribosomal protein L5
BACT000035	*rplF*	50S ribosomal protein L6
BACT000036	*rplL*	50S ribosomal protein L7/L12
BACT000038	*rplI*	50S ribosomal protein L9
BACT000039	*rplJ*	50S ribosomal protein L10
BACT000040	*rplK*	50S ribosomal protein L11
BACT000042	*rplM*	50S ribosomal protein L13
BACT000043	*rplN*	50S ribosomal protein L14
BACT000044	*rplO*	50S ribosomal protein L15
BACT000045	*rplP*	50S ribosomal protein L16
BACT000046	*rplQ*	50S ribosomal protein L17
BACT000047	*rplR*	50S ribosomal protein L18
BACT000048	*rplS*	50S ribosomal protein L19
BACT000049	*rplT*	50S ribosomal protein L20
BACT000050	*rplU*	50S ribosomal protein L21
BACT000051	*rplV*	50S ribosomal protein L22
BACT000052	*rplW*	50S ribosomal protein L23
BACT000053	*rplX*	50S ribosomal protein L24
BACT000056	*rpmA*	50S ribosomal protein L27
BACT000057	*rpmB*	50S ribosomal protein L28
BACT000058	*rpmC*	50S ribosomal protein L29
BACT000059	*rpmD*	50S ribosomal protein L30
BACT000060	*rpmE*	50S ribosomal protein L31
BACT000061	*rpmF*	50S ribosomal protein L32
BACT000062	*rpmG*	50S ribosomal protein L33
BACT000063	*rpmH*	50S ribosomal protein L34
BACT000064	*rpmI*	50S ribosomal protein L35
BACT000065	*rpmJ*	50S ribosomal protein L36

Supplementary Data Table 3: Summary of the 53 loci defined by the ribosomal multilocus sequence typing (rMLST) scheme including the gene name and the protein product.

Species	Number of genomes in comparison dataset per species (including type strain)	Range of ANI values (OrthoANIu) calculated for the green zone (%)	Highest observed ANI value (OrthoANIu) for a genomic match with a different species (%)	Range of ANI values (FastANI) calculated for the green zone (%)	Highest observed ANI value (FastANI) for a genomic match with a different species (%)
Klebsiella aerogenes	292	100 - 95.76	85.67	100 - 95.73	86.72
Klebsiella africana	2	100 - 99.38	95.55	100 - 99.37	95.53
Klebsiella grimontii	97	100 - 98.92	96.12	100 - 98.92	96.10
Klebsiella huaxiensis	3	100 - 99.16	91.59	100 - 99.12	92.21
Klebsiella indica	1	100 - 100	87.03	100 - 100	86.55
Klebsiella michiganensis	224	100 - 97.48	94.00	100 - 97.37	94.11
Klebsiella oxytoca	162	100 - 98.96	92.51	100 - 98.94	92.63
Klebsiella pasteurii	13	100 - 99.25	96.24	100 - 99.21	96.26
Klebsiella pneumoniae	9,766	100 - 97.93	95.55	100 - 97.91	95.47
Klebsiella quasipneumoniae	587	100 - 95.83	95.06	100 - 95.67	95.00
Klebsiella quasivariicola	14	100 - 98.99	95.26	100 - 98.90	95.33
Klebsiella spallanzanii	4	100 - 97.94	91.48	100 - 98.03	92.13
Klebsiella variicola	431	100 - 96.96	95.15	100 - 96.87	95.09
Raoultella electrica	1	100 - 100	94.16	100 - 100	94.04
Raoultella ornithinolytica	80	100 - 99.25	96.13	100 - 99.11	96.00
Raoultella planticola	37	100 - 99.14	96.11	100 - 98.94	95.94
Raoultella terrigena	36	100 - 96.85	85.64	100 - 96.64	86.23

Supplementary Data Table 4: Summary of observed Average Nucleotide Identity (ANI) values calculated by OrthoANIu and FastANI software for 11,750 genomic comparisons against each of the 17 *Klebsiella/Raoultella* type strain genomes (rounded to 2 decimal places). The green zone range of ANI values observed between genomic matches of the same species is typically between 100% and 96%. The highest observed ANI value for a genomic comparison with a different species is 96% or below.

Species	Number of genomes in comparison dataset per species (including type strain)	Range of rMLST-NI values calculated for the green zone (%)	Highest observed rMLST-NI value for a genomic match with a different species (%)
Klebsiella aerogenes	292	100 – 99.856	98.240
Klebsiella africana	2	100 – 99.952	99.757
Klebsiella grimontii	97	100 – 99.885	99.698
Klebsiella huaxiensis	3	100 – 99.971	98.006
Klebsiella indica	1	100 – 100.000	97.465
Klebsiella michiganensis	224	100 – 99.823	99.640
Klebsiella oxytoca	162	100 – 99.904	99.463
Klebsiella pasteurii	13	100 – 99.947	99.703
Klebsiella pneumoniae	9,766	100 – 99.780	99.727
Klebsiella quasipneumoniae	587	100 – 99.837	99.741
Klebsiella quasivariicola	14	100 – 99.957	99.746
Klebsiella spallanzanii	4	100 – 99.808	98.715
Klebsiella variicola	431	100 – 99.631	99.216
Raoultella electrica	1	100 – 100.000	99.573
Raoultella ornithinolytica	80	100 – 99.956	99.808
Raoultella planticola	37	100 – 99.966	99.813
Raoultella terrigena	36	100 – 99.593	98.260

Supplementary Data Table 5: Summary of observed rMLST nucleotide identity (rMLST-NI) values for 11,750 genomic comparisons against each of the 17 *Klebsiella/Raoultella* type strain genomes (rounded to 3 decimal places). The green zone range of rMLST-NI values observed between genomic matches of the same species is typically between 100% and 99.8%. The highest observed rMLST-NI value for a genomic comparison with a different species is 99.8% or below.

Number of CPU Cores	OrthoANIu	FastANI	rMLST Nucleotide Identity (MLSS.pl)	Kleborate Species Scan (MASH)
1	84,651s (23h 30m 51s)	235s (3m 55s)	52s (0m 52s)	179s (2m 59s)
10	9,457s (2h 37m 37s)	45s (0m 45s)	n/a	n/a
20	5,442s (1h 30m 42s)	31s (0m 31s)	n/a	n/a

Supplementary Data Table 6: Time taken to scan 17 type strain genomes against each library. The OrthoANIu and FastANI libraries contain 17 type strain genomes. The rMLST-NI library contains 618 rMLST allele DNA sequences and 17 rMLST profiles. The Kleborate k-mer sketch library contains 2,620 entries. OrthoANIu and FastANI can be configured to run on multiple CPU cores, whilst the rMLST-NI method (using MLSS.pl) and Kleborate species scan run on one CPU core.

Isolate Id	Isolate Name	NCBI Assembly Identifier (Genbank)	Species of Best OrthoANIu Match (Average Nucleotide Identity)	Species in Source Database (Average Nucleotide Identity)
7586	NCTC9644	GCA_900636315.1	*K. pneumoniae* (99.1%)	*K. aerogenes* (84.9%)
8778	4928STDY7071692	GCA_902163325.1	*K. pasteurii* (99.4%)	*K. grimontii* (96.0%)
8779	4928STDY7071693	GCA_902163335.1	*K. pasteurii* (99.5%)	*K. grimontii* (95.9%)
8780	4928STDY7071694	GCA_902163315.1	*K. pasteurii* (99.4%)	*K. grimontii* (96.1%)
8781	4928STDY7071695	GCA_902163345.1	*K. pasteurii* (99.5%)	*K. grimontii* (96.0%)
8816	SPARK1531C2	GCA_902158675.1	*K. pasteurii* (99.3%)	*K. grimontii* (96.2%)
11537	1001295B_180824_F3	GCA_015550565.1	*K. pasteurii* (99.4%)	*K. grimontii* (95.9%)
52	SA2	GCA_000427015.1	*K. grimontii* (99.4%)	*K. michiganensis* (93.8%)
337	1169_SBOY	GCA_001060405.1	*K. grimontii* (99.2%)	*K. michiganensis* (93.8%)
412	1037_KOXY	GCA_001053665.1	*K. grimontii* (99.2%)	*K. michiganensis* (93.8%)
421	1154_KOXY	GCA_001052825.1	*K. grimontii* (99.2%)	*K. michiganensis* (93.7%)
431	371_KOXY	GCA_001054995.1	*K. grimontii* (99.3%)	*K. michiganensis* (93.8%)
432	375_KOXY	GCA_001070955.1	*K. grimontii* (99.3%)	*K. michiganensis* (93.9%)
433	397_KOXY	GCA_001072735.1	*K. grimontii* (99.3%)	*K. michiganensis* (93.8%)
434	409_KOXY	GCA_001072835.1	*K. grimontii* (99.2%)	*K. michiganensis* (93.8%)
441	636_KOXY	GCA_001065765.1	*K. grimontii* (98.9%)	*K. michiganensis* (93.8%)
442	649_KOXY	GCA_001066775.1	*K. grimontii* (99.4%)	*K. michiganensis* (93.8%)
775	452_SSON	GCA_001076805.1	*K. grimontii* (99.2%)	*K. michiganensis* (93.8%)
819	M5al	GCA_001633115.1	*K. grimontii* (99.5%)	*K. michiganensis* (93.8%)
820	SA2	GCA_000733495.1	*K. grimontii* (99.4%)	*K. michiganensis* (93.7%)
3400	D14	GCA_002856195.1	*K. grimontii* (99.2%)	*K. michiganensis* (93.7%)
9226	MGYG-HGUT-00093	GCA_902363155.1	*K. grimontii* (99.3%)	*K. michiganensis* (93.7%)
47	10-5250	GCA_000247915.1	*K. pasteurii* (99.4%)	*K. michiganensis* (93.9%)
438	625_KOXY	GCA_001057685.1	*K. pasteurii* (99.5%)	*K. michiganensis* (93.9%)
439	628_KOXY	GCA_001065705.1	*K. pasteurii* (99.5%)	*K. michiganensis* (93.9%)
1847	PO2731	GCA_002186735.1	*K. pasteurii* (99.4%)	*K. michiganensis* (93.8%)
9843	ARO112	GCA_009757395.1	*K. pasteurii* (99.5%)	*K. michiganensis* (93.7%)
10756	CRN 17	GCA_013266985.1	*K. pasteurii* (99.5%)	*K. michiganensis* (93.9%)
11908	132656-17	GCA_015679345.1	*K. pasteurii* (99.6%)	*K. michiganensis* (94.0%)
11925	KLO00002	GCA_015721765.1	*K. pasteurii* (99.1%)	*K. michiganensis* (93.9%)
12257	B106	GCA_016616645.1	*K. pasteurii* (99.4%)	*K. michiganensis* (93.9%)
12261	KA98_C	GCA_016652955.1	*K. pasteurii* (99.5%)	*K. michiganensis* (93.8%)
420	1148_KOXY	GCA_001052235.1	*K. grimontii* (99.2%)	*K. oxytoca* (91.4%)
1723	JKo3	GCA_001548355.1	*K. grimontii* (99.2%)	*K. oxytoca* (91.4%)
1852	DSM 29614	GCA_002080105.1	*K. grimontii* (99.2%)	*K. oxytoca* (91.5%)
4285	NK-1	GCA_003339485.1	*K. grimontii* (99.3%)	*K. oxytoca* (91.4%)
4976	SN1015-66	GCA_003416995.1	*K. grimontii* (99.1%)	*K. oxytoca* (91.4%)
4977	H6	GCA_003417005.1	*K. grimontii* (99.2%)	*K. oxytoca* (91.4%)
8734	4928STDY7071416	GCA_902160405.1	*K. grimontii* (99.2%)	*K. oxytoca* (91.5%)
8735	4928STDY7071417	GCA_902160375.1	*K. grimontii* (99.2%)	*K. oxytoca* (91.5%)
11258	RHBSTW-00432	GCA_013737895.1	*K. grimontii* (99.4%)	*K. oxytoca* (91.5%)
11259	RHBSTW-00452	GCA_013737205.1	*K. grimontii* (99.1%)	*K. oxytoca* (91.5%)
11261	RHBSTW-00651	GCA_013733775.1	*K. grimontii* (99.1%)	*K. oxytoca* (91.5%)
4284	67	GCA_003201885.1	*K. huaxiensis* (95.3%)	*K. oxytoca* (87.2%)
1722	CAV1374	GCA_001022195.1	*K. michiganensis* (98.9%)	*K. oxytoca* (92.3%)
1724	KONIH1	GCA_000714655.1	*K. michiganensis* (98.5%)	*K. oxytoca* (92.3%)
1851	TDB-1	GCA_002111445.1	*K. michiganensis* (98.8%)	*K. oxytoca* (92.2%)
3188	CAV1752	GCA_001970835.1	*K. michiganensis* (98.7%)	*K. oxytoca* (92.3%)
3189	AR_0147	GCA_002072655.1	*K. michiganensis* (98.8%)	*K. oxytoca* (92.4%)
3407	AR_0028	GCA_002947505.1	*K. michiganensis* (98.7%)	*K. oxytoca* (92.3%)
3408	KONIH2	GCA_002906415.1	*K. michiganensis* (98.8%)	*K. oxytoca* (92.3%)

3409	KONIH4	GCA_002906395.1	*K. michiganensis* (98.5%)	*K. oxytoca* (92.3%)
3410	KONIH5	GCA_002906435.1	*K. michiganensis* (98.5%)	*K. oxytoca* (92.3%)
4278	KONIH8	GCA_002918635.1	*K. michiganensis* (98.5%)	*K. oxytoca* (92.3%)
4279	KONIH3	GCA_002918655.1	*K. michiganensis* (98.5%)	*K. oxytoca* (92.3%)
4281	KONIH6	GCA_002918695.1	*K. michiganensis* (98.5%)	*K. oxytoca* (92.4%)
4282	KONIH7	GCA_002919605.1	*K. michiganensis* (98.5%)	*K. oxytoca* (92.3%)
4283	KONIH9	GCA_002919625.1	*K. michiganensis* (98.5%)	*K. oxytoca* (92.3%)
11046	RHBSTW-00493	GCA_013784845.1	*K. michiganensis* (99.1%)	*K. oxytoca* (92.4%)
11256	RHBSTW-00365	GCA_013751295.1	*K. michiganensis* (98.9%)	*K. oxytoca* (92.3%)
11260	RHBSTW-00529	GCA_013747515.1	*K. michiganensis* (99.1%)	*K. oxytoca* (92.3%)
4317	TUM1814	GCA_003176335.1	*K. quasipneumoniae* (96.6%)	*K. pneumoniae* (93.8%)
4731	4300STDY6636983	GCA_900493045.1	*K. quasipneumoniae* (96.5%)	*K. pneumoniae* (93.9%)
6959	KPN1344	GCA_003815095.1	*K. quasipneumoniae* (99.1%)	*K. pneumoniae* (93.6%)
7018	NIT-KP	GCA_003711965.1	*K. quasipneumoniae* (96.6%)	*K. pneumoniae* (93.9%)
7742	GEO_33_Down_A	GCA_004024245.1	*K. quasipneumoniae* (96.6%)	*K. pneumoniae* (93.8%)
7743	GEO_28_Up_A	GCA_004024275.1	*K. quasipneumoniae* (96.5%)	*K. pneumoniae* (93.7%)
11107	KAM260	GCA_013426775.1	*K. quasipneumoniae* (96.6%)	*K. pneumoniae* (93.7%)
11680	INF291-sc-2280225	GCA_904864575.1	*K. quasipneumoniae* (96.7%)	*K. pneumoniae* (93.9%)
1784	YH43	GCA_001548315.1	*K. variicola* (97.0%)	*K. pneumoniae* (94.2%)
4313	TUM1382	GCA_003175595.1	*K. variicola* (99.0%)	*K. pneumoniae* (94.4%)
11640	INF022-sc-2279895	GCA_904863055.1	*K. variicola* (97.8%)	*K. pneumoniae* (96.0%)
11644	INF058-sc-2279968	GCA_904863155.1	*K. variicola* (99.0%)	*K. pneumoniae* (94.7%)
11656	INF154-sc-2279998	GCA_904866265.1	*K. variicola* (98.6%)	*K. pneumoniae* (95.0%)
11667	INF208-sc-2280076	GCA_904864495.1	*K. variicola* (98.6%)	*K. pneumoniae* (95.0%)
11671	INF232-sc-2280123	GCA_904866545.1	*K. variicola* (98.7%)	*K. pneumoniae* (94.9%)
11679	INF290-sc-2280223	GCA_904865775.1	*K. variicola* (98.6%)	*K. pneumoniae* (95.0%)
11695	INF336-sc-2280147	GCA_904866525.1	*K. variicola* (98.7%)	*K. pneumoniae* (95.1%)
11698	INF345-sc-2280164	GCA_904864455.1	*K. variicola* (99.1%)	*K. pneumoniae* (94.5%)
11749	KSB1_10B-sc-2280232	GCA_904866515.1	*K. variicola* (99.2%)	*K. pneumoniae* (94.5%)
11759	KSB1_8D-sc-2280250	GCA_904863415.1	*K. variicola* (98.6%)	*K. pneumoniae* (95.0%)
11760	KSB1_8J-sc-2280307	GCA_904866185.1	*K. variicola* (99.1%)	*K. pneumoniae* (94.5%)
11761	KSB1_9F-sc-2280270	GCA_904865705.1	*K. variicola* (99.0%)	*K. pneumoniae* (94.5%)
11767	KSB2_3A-sc-2280319	GCA_904863065.1	*K. variicola* (99.1%)	*K. pneumoniae* (94.5%)
8616	TUM14061	GCA_004312065.1	*R. electrica* (99.1%)	*R. ornithinolytica* (93.9%)
12242	LL1	GCA_014764465.1	*R. electrica* (98.8%)	*R. ornithinolytica* (94.1%)
1789	S12	GCA_000829965.1	*R. terrigena* (99.1%)	*R. ornithinolytica* (85.4%)
8614	JUb54	GCA_004341525.1	*R. terrigena* (97.3%)	*R. ornithinolytica* (85.3%)
11849	STW0522-56	GCA_015135635.1	*R. electrica* (99.0%)	*R. planticola* (93.5%)

Supplementary Data Table 7: Summary of OrthoANIu results for 89 NCBI Assembly entries where the source database species annotation does not correspond with the closest *Klebsiella/Raoultella* type strain using ANI as the genomic comparison metric. The ANI value for the closest type strain species and the source database species are included.

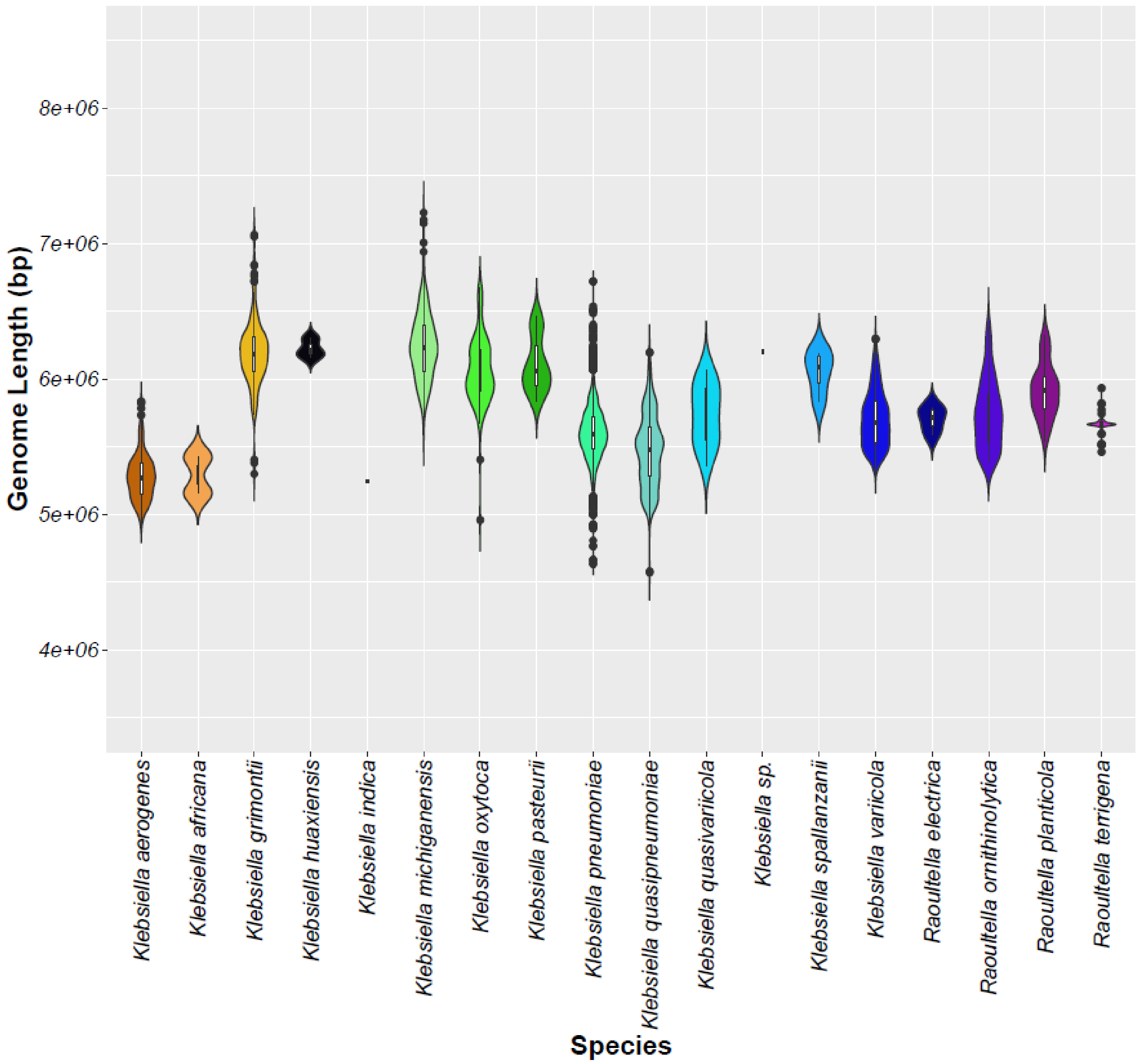

Supplementary Data Figure 1: Graph of genome size ranges of 17 *Klebsiella/Raoultella* species across a dataset of 11,791 genomes. Forty-eight genomes with suspected DNA contamination identified by rMLST allelic matches to non-*Klebsiella/Raoultella* species were excluded from the dataset. The violin plot representation indicates the variation of the genome size within each species.

Provenance/meta data

locus: BACT000035 (rplF)

allele: 2393

sequence: ATGTCTCGTG TTGCTAAAGC ACCGGTCGTT GTTCCTGCCG GCGTTGACGT TAAAGTCAAC GGTCAGGTTA TTACGATCAA AGGTAAAAAT GGCGAGCTGA CTCGTACTCT CAACGATGCT GTTGAAGTTA AACATGCAGA TAATGCTCTG ACCTTCGGTC CGCGTGATGG TTACGTAGAC GGATGGGCTC AGGCTGGTAC CGCGCGTGCC CTGCTGAACT CAATGGTTAT CGGTGTTACC GAAGGCTTCA CTAAAAAGCT TCAGCTGGTT GGTGTAGGCT ATCGTGCAGC GGTTAAAGGG GATGTAGTAA ACCTGGCTTT AGGTTTCTCT CATCCTGTTG AACATAAGCT GCCGGCCGGT ATCACTGCAG AATGCCCGAC TCAAACTGAA ATCGTGCTGA AAGGCGCTGA TAAGCAGGTG ATCGGTCAGG TTGCAGCAGA TCTGCGCGCC TACCGTCGTC CTGAGCCTTA CAAAGGCAAG GGTGTTCGTT ACGCCGACGA AGTCGTGCGT ACCAAAGAGG CTAAGAAGAA GTAA

length: 534

status: WGS: automated extract (BIGSdb)

date entered: 2014-01-03

datestamp: 2014-01-03

sender: James Bray

curator: James Bray, University of Oxford, UK

Supplementary Data Figure 2: An example allelic entry of the BACT000035/rplF gene from the rMLST sequence definition database hosted on the PubMLST website (https://pubmlst.org/species-id). This rMLST allele is number 2393 and is 534 base pairs in length. This allele is found in some K*lebsiella aerogenes* isolates.

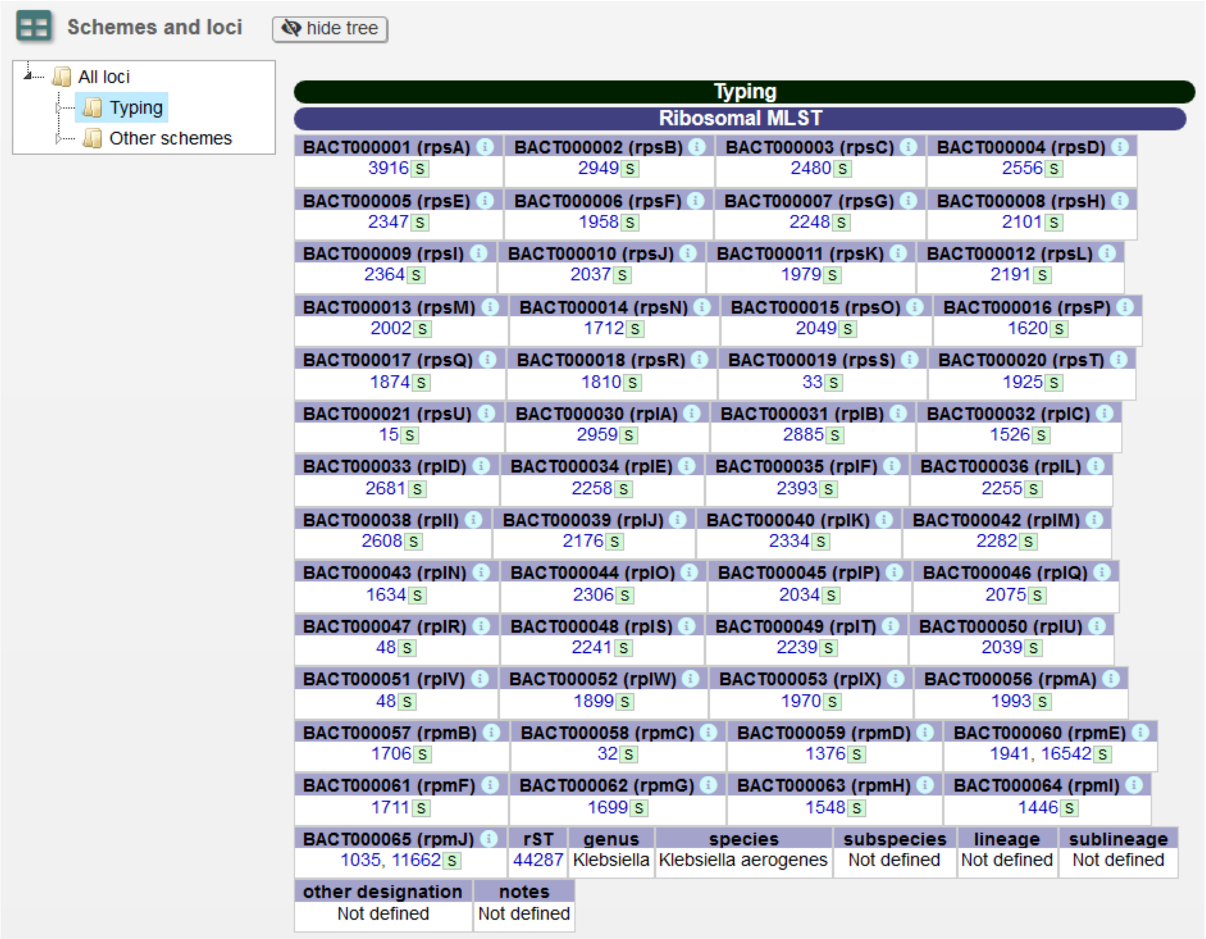

Schemes and loci   hide tree

All loci
Typing
Other schemes

Typing

Ribosomal MLST

Locus	Allele
BACT000001 (rpsA)	3916
BACT000002 (rpsB)	2949
BACT000003 (rpsC)	2480
BACT000004 (rpsD)	2556
BACT000005 (rpsE)	2347
BACT000006 (rpsF)	1958
BACT000007 (rpsG)	2248
BACT000008 (rpsH)	2101
BACT000009 (rpsI)	2364
BACT000010 (rpsJ)	2037
BACT000011 (rpsK)	1979
BACT000012 (rpsL)	2191
BACT000013 (rpsM)	2002
BACT000014 (rpsN)	1712
BACT000015 (rpsO)	2049
BACT000016 (rpsP)	1620
BACT000017 (rpsQ)	1874
BACT000018 (rpsR)	1810
BACT000019 (rpsS)	33
BACT000020 (rpsT)	1925
BACT000021 (rpsU)	15
BACT000030 (rplA)	2959
BACT000031 (rplB)	2885
BACT000032 (rplC)	1526
BACT000033 (rplD)	2681
BACT000034 (rplE)	2258
BACT000035 (rplF)	2393
BACT000036 (rplL)	2255
BACT000038 (rplI)	2608
BACT000039 (rplJ)	2176
BACT000040 (rplK)	2334
BACT000042 (rplM)	2282
BACT000043 (rplN)	1634
BACT000044 (rplO)	2306
BACT000045 (rplP)	2034
BACT000046 (rplQ)	2075
BACT000047 (rplR)	48
BACT000048 (rplS)	2241
BACT000049 (rplT)	2239
BACT000050 (rplU)	2039
BACT000051 (rplV)	48
BACT000052 (rplW)	1899
BACT000053 (rplX)	1970
BACT000056 (rpmA)	1993
BACT000057 (rpmB)	1706
BACT000058 (rpmC)	32
BACT000059 (rpmD)	1376
BACT000060 (rpmE)	1941, 16542
BACT000061 (rpmF)	1711
BACT000062 (rpmG)	1699
BACT000063 (rpmH)	1548
BACT000064 (rpmI)	1446
BACT000065 (rpmJ)	1035, 11662

rST	genus	species	subspecies	lineage	sublineage
44287	Klebsiella	Klebsiella aerogenes	Not defined	Not defined	Not defined

other designation	notes
Not defined	Not defined

Supplementary Data Figure 3: rMLST allele designations for all 53 rMLST loci of the *Klebsiella aerogenes* type strain isolate KCTC 2190 (*Klebsiella* project database isolate id 23).

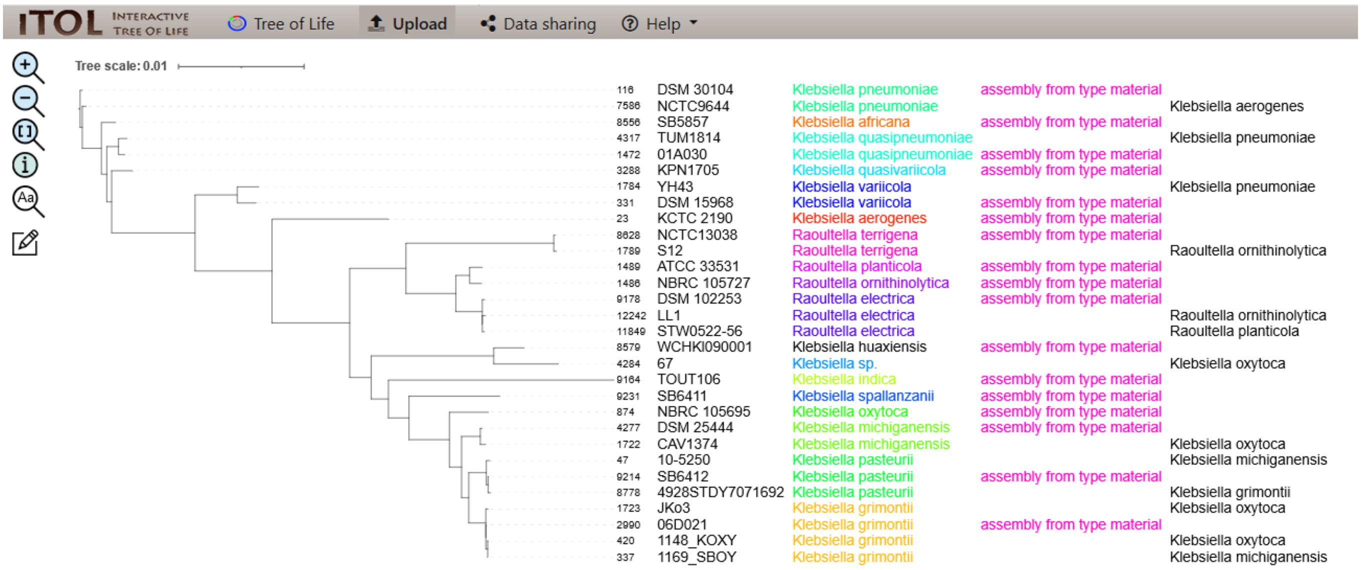

Supplementary Data Figure 4: Interactive Tree of Life (iTOL, https://itol.embl.de) visualisation of a phylogenetic tree of 30 *Klebsiella/Raoultella* isolates including 17 type strains and 13 non-type strain isolates where the NCBI Assembly species annotation was not consistent with the automated species identification scan results. The neighbour-joining tree was calculated by CLUSTAL W (v2.1) from the concatenated alleles of non-paralogous rMLST loci. Each isolate is annotated with (1) database identifier, (2) isolate name (3) species (type strain and predicted species), (4) ‘assembly from type material’ if isolate is a type strain (5) species in source database (if different). This tree was constructed in 12 seconds on the *Klebsiella* project database from existing rMLST allele designations and DNA sequences stored in the rMLST sequence definition database.

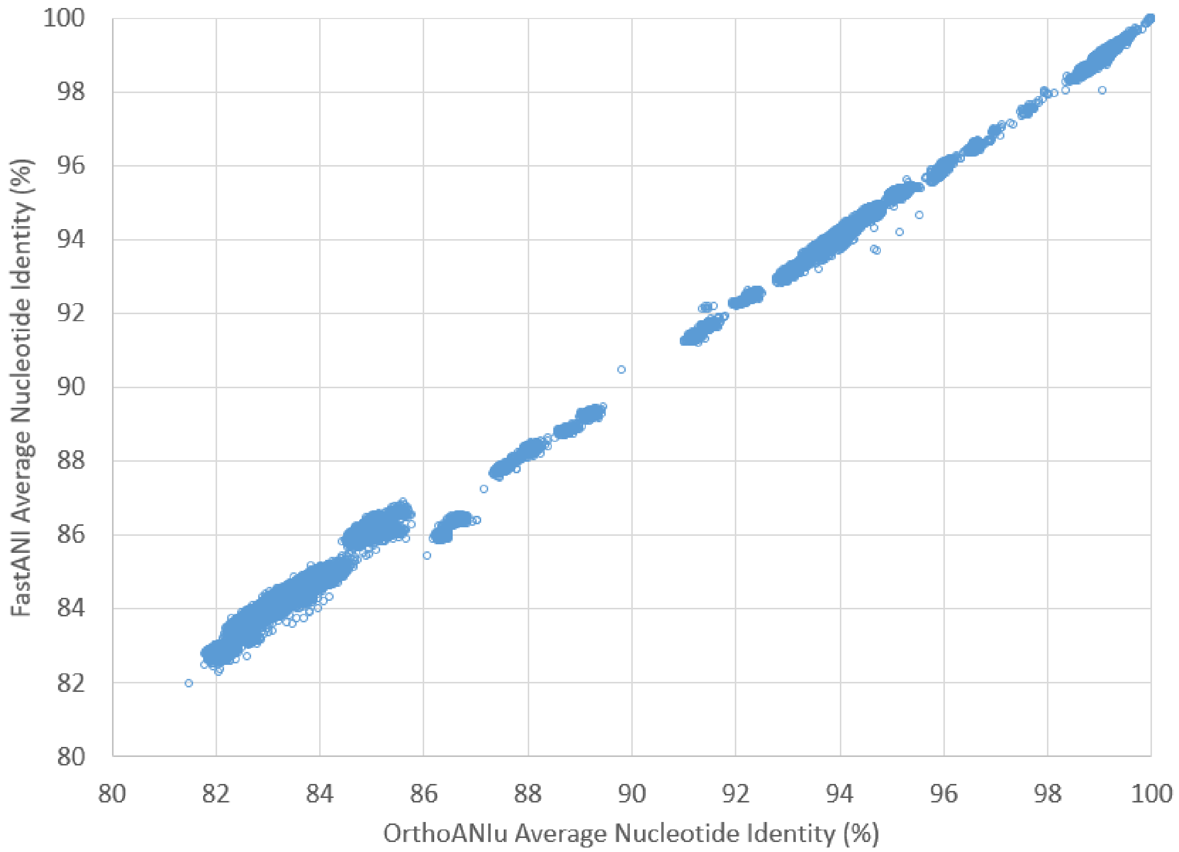

Supplementary Data Figure 5: Comparison of OrthoANIu average nucleotide identity (ANI) values with FastANI ANI values for 11,839 genomes against the library of 17 *Klebsiella/Raoultella* type strain genomes (201,263 pairwise comparisons for each algorithm). The R^2 value is 0.999.
